# Improved Intelligent Condition Monitoring with Diagnostic Indicator Selection

**DOI:** 10.3390/s25010137

**Published:** 2024-12-29

**Authors:** Urszula Jachymczyk, Paweł Knap, Krzysztof Lalik

**Affiliations:** Faculty of Mechanical Engineering and Robotics, AGH University of Krakow, 30-059, Krakow, Poland; delhi@student.agh.edu.pl (U.J.); klalik@agh.edu.pl (K.L.)

**Keywords:** predictive maintenance, condition indicators, artificial intelligence, feature selection, vibrodiagnostics, fault identification

## Abstract

In this study, a predictive maintenance (PdM) system focused on feature selection for the detection and classification of simulated defects in wind turbine blades has been developed. Traditional PdM systems often rely on numerous, broadly chosen diagnostic indicators derived from vibration data, yet many of these features offer little added value and may even degrade model performance. General feature selection methods might not be suitable for PdM solutions, as information regarding observed faults is often misinterpreted or lost. To address these issues, a structured feature selection method based on correlation analysis supplemented with comprehensive visual evaluation was proposed. Unlike generic dimensionality reduction techniques, this approach preserves critical domain-specific information and avoids misinterpretation of fault indicators. By applying the proposed method, it was possible to successfully filter out redundant features, enabling simpler machine learning (ML) models to match or even surpass the performance of more complex deep learning (DL) architectures. The best results were achieved by a deep neural network trained on the full dataset, with accuracy, precision, recall, and F1 score of 97.30%, 97.23%, 97.23%, and 97.23%, respectively, while the top-performing ML model (a voting classifier trained on the reduced dataset) attained scores of 97.13%, 96.99%, 96.95%, and 96.94%. The proposed method for reducing condition indicators successfully decreased their number by approximately 3.27 times, simultaneously significantly reducing computational time of prediction, reaching up to 50% reduction for complex models. In doing so, we lowered computational demands and improved classification efficiency without compromising accuracy for ML models. Although feature reduction did not similarly benefit the metrics for DL models, these findings highlight that well-chosen, domain-relevant condition indicators can streamline data input and deliver interpretable, cost-effective PdM solutions suitable for industrial applications.

## 1. Introduction

The dynamic development of the manufacturing industry resulted in necessary implementation of intelligent technologies for condition monitoring of industrial machinery [1,2]. The condition monitoring market is experiencing consistent growth, with projections indicating an increase from USD 2.6 billion in 2019 to USD 3.9 billion by 2025 [3]. An effective strategy is *Predictive Maintenance* (PdM) [4], which allows for optimal utilization of equipment, saving costs associated with *preventive maintenance* (an approach of systematic inspections and replacement of machinery parts before they show signs of wear and run into failure), while also preventing downtime through well-planned service work. PdM systems fulfill three key tasks: detecting faults, classifying them, and predicting time of failures.

PdM systems allows for the prediction of potential failures and the planning of service actions based on sensor data (e.g., vibrations, sound, temperature, pressure, and many others). Vibration analysis is widely regarded as superior in condition monitoring and damage detection tasks, as it offers an immediate response to changes and can be employed for both intermittent and continuous monitoring [5]. Various signal processing techniques combined with statistical methods are used to extract significant information from vibration signals [6,7]. In many cases, improvements are sought by incorporating new condition indicators [8]. However, an increasing number of condition indicators can introduce complexity for AI-based (artificial intelligence-based) algorithms, as not all data may be relevant for effective monitoring, and excessive input may even degrade performance.

A noticeable trend is the usage of neural networks in PdM systems, which take almost unprocessed data and achieve satisfactory results at the same time. The lack of analysis may result in worse understanding of the process and lack of essential knowledge about the relevance of individual condition indicators (CI) when creating future systems. Therefore, this research is determined to prove that for PdM, similar results can be achieved by using less complicated machine learning (ML) algorithms.

One of the current challenges in AI-based predictive maintenance is model selection [9]. The same iterative approach applies to the selection of condition indicators. The excessive number of CIs may result in a so-called *curse of dimensionality*, which is the risk associated with large numbers of dimensions. It causes the prediction to be less reliable; because points in the dataset are very sparse, the model might overfit the training data [10].

This research explores the relationships between Feature Selection (FS), interpretability, and computational efficiency. By evaluating the impact of feature reduction on algorithm performance, we aim to optimize industrial PdM systems through the development of a method that is both computationally efficient and capable of ensuring precise control while preserving essential domain-specific knowledge. The results will be compared based on the task of detection and classification of anomalous operation of rotating devices.

### 1.1. Condition Monitoring in Wind Turbines

Monitoring machine conditions is crucial for equipment responsible for significant tasks, such as generators and pumps. An example of this kind of device can be wind turbines, where an essential part of the total costs of electricity generation in wind power is the service costs of the turbines. These expenditures include insurance, regular maintenance, repairs, spare parts, and administration and are estimated to account for 20 to 25% of the total turbine maintenance costs [11]. Hence, the total cost of wind energy generation is noticeably higher compared to the costs of generation in coal and gas power plants [12], and it is necessary to introduce new diagnostic techniques. Among the most common damages encountered in wind farms, the following can be distinguished:

- damage to the generator,
- damage to the wind turbine blades,
- damage to the wind turbine tower.

Among the various wind turbine failures presented in [13], rotating parts (including bearings and blades) are the most prone to mechanical damage and are usually detected with a delay. These defects typically arise from prolonged and changing loads, material fatigue, manufacturing errors, erosion, light and ultraviolet radiation, icing, penetration of oils causing delamination (pitting), or frequency resonance [14]. In the case of blades, defects are additionally caused by collisions with objects and lightning strikes. The most common recorded cause of damage was the detachment of fragments or entire blades from the rotor. By 31 March 2022, a total of 499 cases had been registered, accounting for 15.46% of all incidents [15].

One of the most commonly used diagnostic signals in PdM systems are vibrations, which carry extensive information about the technical state of the equipment and indicate potential issues. In an ideal situation, machines would not generate any vibrations because all supplied energy would be completely converted into useful work. However, in reality, components constantly interact with each other, and some energy is dissipated in the form of vibrations. All changes resulting from component wear, errors in the interaction of individual elements, clearances, and misalignment are reflected in the increase in vibration energy, which, when dissipated in the machine, excites resonances and significantly increases dynamic loads. Therefore, cause and effect interact, driving each other [16].

The wide range of possible data processing paths within vibration analysis leads to a diversity of measurement technologies, potential diagnostic indicators, visualization methods, and resultative architectures of PdM systems [17]. For diagnostics, except for vibration analysis, additional auxiliary signals such as temperature or current are used. However, vibrations are comprehensive and sufficient tools for the detection and classification of machine damage [18], so this work focuses solely on them.

### 1.2. Artificial Intelligence in PdM

As early as 2001, a highly influential article by Michele Banko and Eric Brill [19] demonstrated that various algorithms, both simple and more complex, achieve similar results if they are trained on a sufficiently large amount of good input data. The authors summarized the results with the following words: “These results suggest that we may want to reconsider the trade-off between spending time and money on algorithm development versus spending it on corpus development”. In line with this quote, the aim of the experiment is to compare the performance of algorithms of varying complexity on a classification task of fault detection of rotating devices.

Based on studies conducted by [20], the number of articles related to PdM published between 2009 and 2018 has increased significantly (using the extraction criteria of [21]). The average number of papers increased from 0.5 article per year in 2009–2012 to 11.3 articles per year in 2013–2018. The authors of [21] discern the reason for this phenomenon in the amount of data generated by industrial equipment and, correlated with that, advances in ML algorithms. According to [22], in 2020, the number of articles directly related to PdM and ML that were published in one of the five most known electronic sources, covering the concepts of Industry 4.0. after the year 2015, amounted to 155.

Diagnostic approaches in condition monitoring can generally be grouped into two main categories: those relying on extracted features and those using raw time-series data. In the context of PdM, ML algorithms require preprocessing raw data to derive feature matrices, which are then used as inputs for classifiers. For example, ref. [23] used a Support Vector Machine (SVM) combined with the binary particle swarm optimization algorithm for FS, focusing on maximizing class separability by incorporating the regularized Fisher’s criterion to detect bearing faults. SVMs can also be used for bearings’ Remaining Useful Life (RUL) calculations, as shown in [24], where principal component analysis (PCA) was also used to reduce the number of features. Decision trees can also be used to provide autonomous diagnostic solutions [25]. The authors of [26] used a method based on C4.5 decision tree and PCA to classify six faults for the Bently Rotor Kit RK4. The Random Forest (RF) approach is widely used in PdM solutions. It has been tested on real industrial data [27] and a real-time predictive fault detection system of a hard disk [28]. Both studies achieved high accuracy and proved great potential in industrial solutions. In [18], RF and XGBoost models were evaluated, comparing vibration and current input data, achieving remarkably high accuracy, nearly 100% for classifying different health states of induction motors (IMs), outperforming deep learning (DL) models. For PdM tasks, K-Nearest Neighbor (KNN) is widely used, which is especially used for fault diagnosis of rolling element bearings [29], gears and gearboxes [30], and motors [31]. Also, naïve Bayesian [32] and Autoregressive Integrated Moving Average (ARIMA) [33,34] find widespread application in this context.

The application of deep learning (DL) to PdM has grown rapidly, with the number of related publications increasing from nearly zero in 2014 to almost 2500 in 2021 [35]. This surge in interest stems from the ability of DL methods to directly learn from raw data, thereby overcoming certain limitations of traditional ML approaches that rely heavily on expert-driven feature extraction and selection. For example, feedforward neural networks, such as Multilayer Perceptrons (MLPs) [36], have been employed in order to improve maintenance schedules [37,38]. Convolutional Neural Networks (CNNs) [39] have been adapted for vibration- and image-based condition monitoring tasks, demonstrating strong capabilities in automatically identifying patterns linked to potential equipment failures [40,41]. The authors of [18] compared the results of 1D (one dimensional) and 2D (two dimensional) CNNs trained on raw data for monitoring the health IMs. Similarly, Autoencoders (AEs) [42] have gained traction for anomaly detection in PdM applications by learning compact representations that encapsulate normal operating conditions and flagging deviations [43,44,45]. When working with time-series data, Recurrent Neural Networks (RNNs) [46] have proven effective for capturing temporal dependencies [47,48]. Moreover, Long Short-Term Memory (LSTM) networks [49] have been employed to address vanishing gradient issues and handle longer-term dependencies [50,51].

### 1.3. Research Gap

FS techniques in PdM are mostly used to prevent the curse of dimensionality and improve the effectiveness of models by selecting only the best indicators. FS methods can be broadly classified into three categories:

- filter—selecting features based on statistical measures or ranking without involving a learning algorithm,
- wrapper—using a learning algorithm to evaluate subsets of features and optimize model performance iteratively,
- embedded methods—incorporating FS directly within the training process of a learning algorithm.

In [52], a wide range of filter methods are presented. The most commonly used dimensionality reduction is PCA [24,26,53], which allows one to reduce dimensions but at the same time almost always results in decreases in model performance, as its goal is not to select the best features. Among most state-of-the-art methods encountered in PdM solutions, the following can be distinguished: Binary Particle Swarm Optimization (BPSO) [23] and RF [54,55].

Despite growing interest in PdM, there remains a notable gap in solutions specifically focused on FS. Most methods are employed without proper control over the selected parameters, often applied in an arbitrary manner. We want to propose a method that allows for full control over the number and quality of selected indicators through visual analysis. The correlation coefficient is computationally inexpensive and can be applied to each application according to specific requirements determined by the system. However, inexpensive filter methods are usually based solely on numerical values, without further conclusions. In contrast, BPSO is computationally expensive, as it requires multiple model trainings in each iteration. The cost function is based on the results of a model trained on different feature subsets that change in every iteration of the algorithm. This iterative process can potentially lead to the algorithm becoming stuck in a local minimum. It is extremely important to choose an appropriate cost function, which requires specialist knowledge.

According to [56], general FS methods are suitable for general data but may not be effective for asset data in PdM due to two key reasons: first, the interpretation of feature characteristics differs between general and asset data (e.g., low variance might indicate a nominal state in asset data, while high variance could signal an unreliable sensor); second, standard methods do not account for the retention of domain-specific knowledge. These methods are often criticized for selecting too many redundant features, which can lead to correlated features. Information regarding observed faults is often misinterpreted or lost when general feature engineering is performed on asset data. As a result, standard FS methods may not capture the necessary domain-specific information required in PdM.

While DL models offer promising performance improvements, many studies still focus on generic architectures and almost unprocessed data without thoroughly examining how different types or numbers of features influence diagnostic accuracy. In addition, relatively few works systematically investigate the relationship between FS, model interpretability, and computational efficiency; a crucial consideration for PdM systems in industrial environments. The existing literature suggests a need for more structured methodologies and comparative analyses that tailor both input features and DL architectures to specific types of equipment, data availability, and maintenance objectives. Addressing these gaps can help to establish more standardized approaches to condition monitoring, enabling practitioners to make informed decisions when implementing PdM strategies.

### 1.4. Motivation and Novelty

Modern condition monitoring systems can process hundreds of indicators derived from data collected by numerous sensors [57,58,59]. The vast quantity of data complicates the design of expert-based solutions, making them time-consuming and resource-intensive [60]. In contrast, AI-driven systems can utilize these extensive data to produce diagnostic insights [21]. However, currently, there is no standardized method to ensure that the indicators provided to the AI algorithms for PdM are optimally used [9].

We propose a novel method for FS that ensures both control over the number and quality of selected indicators and preserves domain-specific knowledge critical for PdM applications. This solution addresses the research gap outlined in Section 1.3. By incorporating a correlation coefficient-based approach, our method is computationally inexpensive and adaptable to specific system requirements, enabling practitioners to visually analyze and select features relevant to their use case. This approach mitigates the limitations of purely numerical filter methods while avoiding the high computational costs of wrapper methods.

The motivation behind this work was to develop a method that enables the selection of only the most relevant indicators and to assess the impact of reducing the number of features on algorithm performance. In systems requiring data from a large array of sensors, this approach could substantially lower the computational demands of algorithm operation [61]. Additionally, such a reduction can help identify the sensors that contribute most to algorithm effectiveness, potentially leading to a decrease in the overall number of sensors required. This has two key advantages:

- Economic efficiency: Fewer sensors translate to lower costs.
- Improved CM (condition monitoring) reliability: Fewer sensors reduce the risk of downtime caused by sensor malfunctions, which are challenging to detect and often require human intervention [62]. Furthermore, some sensor data may be redundant from the perspective of the AI-based algorithm, allowing for further optimization.

## 2. Methodology

This section describes the methodology employed in this study, including the calculation of vibration condition indicators in both time and frequency domains and complex signal processing techniques with filtration. A comprehensive overview of the measurement system, where the experiments were conducted, is provided, with emphasis on its critical components and the faults types examined in the propeller blades. Additionally, all deep learning and machine learning algorithms used in the final solution are thoroughly described.

The methodology includes popular vibration CI analysis with a focus on FS, leading to an optimized solution leveraging DL and ML algorithms by filtering out irrelevant features to achieve a more efficient and effective PdM solution.

### 2.1. Condition Indicators

The concept of diagnostic indicators, also known as condition indicators or health indicators, is commonly used to assess the current state of the subject under investigation. In the context of PdM, they refer to parameters used to monitor the technical condition of equipment, machinery, or production systems. PdM systems typically use broad-spectrum diagnostic indicators based on vibration measurements or velocity estimators. They are primarily computationally efficient and are not typically sensitive to disturbances and signal variations. Based on [63], a selection of indicators has been made, which are presented below:

1. Peak-to-peak (PP)—difference between maximum and minimum value of the signal *x*.
  (1) PP=max(x)−min(x)
2. Root Mean Square (RMS)—reflects the vibration amplitude and energy of the signal in the time domain.
  (2) $$\mathrm{RMS}=\sqrt{\frac{1}{N}\cdot \sum_{i=1}^{N}x_{i}^{2}}$$
  where:
  *N*—number of samples,
  $x_{i}$—i-th signal sample.
3. Crest Factor (CF)—peak value divided by the root mean square $RMS_{x}$. The crest factor can provide an early warning for faults when they first develop because they often first manifest themselves in changes in the peakedness of a signal before they manifest in the energy.
  (3) $$\mathrm{CF}=\frac{max(x)}{RMS_{x}}$$
4. Standard Deviation (STD)—measures the amount of variation from the mean $\bar{x}$ of the data set.
  (4) $$\mathrm{STD}=\sqrt{\frac{1}{N-1}\cdot \sum_{i=1}^{N}{\left(x_{i}-\bar{x}\right)}^{2}}$$
5. Kurtosis—provides a measure of the peakedness of the signal. Developing faults can increase the number of outliers and therefore increase the value of the kurtosis metric. It is calculated as the fourth-order normalized moment of a given signal.
  (5) $$K=\frac{N\cdot \sum_{i=1}^{N}{\left(x_{i}-\bar{x}\right)}^{4}}{{\left(\sum_{i=1}^{N}{\left(x_{i}-\bar{x}\right)}^{2}\right)}^{2}}$$
6. Shape Factor (SF)—represents the time series distribution of the signal in the time domain.
  (6) $$\mathrm{SF}=\frac{\sqrt{\frac{1}{N}\cdot \sum_{i=1}^{N}x_{i}^{2}}}{\frac{1}{N}\cdot \sum_{i=1}^{N}\left|x_{i}\right|}$$
7. Mean Frequency (MF)—indicates the vibration energy in the frequency domain.
  (7) $$\mathrm{MF}=\frac{1}{N}\cdot \sum_{i=1}^{N}X_{n}$$
  where:
  $X_{n}$—amplitude of n-th spectral line,
  *N*—total number of spectral lines.
8. Frequency Center (FC)—informs about the dominant frequency or characteristic central frequency of the signal.
  (8) $$\mathrm{FC}=\frac{\sum_{i=1}^{N}f_{n}\cdot X_{n}}{\sum_{i=1}^{N}X_{n}}$$
  where:
  $f_{n}$—frequency of n-th spectral line.
9. Velocity Root Mean Square (VRMS)—velocity CI. The VRMS is a broadband indicator, since the velocity signal is useful in the range from a few Hz to 1 kHz. According to the ISO 20816 standard [64], depending on the nominal rotational speed of the machine, the lower limit is 2 Hz (for speeds above 120 RPM) or 10 Hz (for speeds above 600 RPM). To quickly calculate the velocity spectrum from vibration signals, omega arithmetic (Equation (9)) is used. This method is more efficient than standard integration operations because it is calculated directly in the frequency domain *f* while offering the same results (provided that frequencies close to 0 are excluded, where the algorithm tends towards infinity), as presented in [17]. Then, the root mean square velocity is calculated according to Equation (2).
  (9) $$\int A\cdot \sin\left(2\cdot \pi \cdot f\cdot t\right)\;dt=-A\cdot \frac{1}{2\cdot \pi \cdot f}\cdot \cos\left(2\cdot \pi \cdot f\cdot t\right)$$
  where *A*—amplitude.

### 2.2. Classifier Metrics

After the training process is completed, it is necessary to evaluate the effectiveness of the model. This can involve both visual measures (confusion matrix) and scalar measures (standard deviation, precision, accuracy, etc.). The most commonly used classification metrics include the following:

- Accuracy: the ratio of all correctly identified cases (*true positive*, *TP* and *true negative*, *TN*) to all cases:
  (10) $$\mathrm{accuracy}=\frac{TP+TN}{TP+FP+TN+FN}$$
- precision: the ratio of correctly identified positive cases (*true positive*) to all cases classified as positive (*true positive + false positive*, *FP*). It can be expressed as follows:
  (11) $$\mathrm{precision}=\frac{TP}{TP+FP}$$
- recall: the ratio of correctly identified positive cases (*True Positive*) to all actual positive cases (*True Positive + False Negative*, *FN*). It can be expressed as:
  (12) $$\mathrm{recall}=\frac{TP}{TP+FN}$$
- F1 score: combination of the two above measures. It is calculated as the harmonic mean between precision and recall:
  (13) $$F1=\frac{2}{\frac{1}{precision}+\frac{1}{recall}}=2\cdot \frac{precision\cdot recall}{precision+recall}=\frac{TP}{TP+\frac{FN+FP}{2}}$$
- Confusion matrix: A confusion matrix is a table that displays the number of correct and incorrect classifications for each class. While it is not a direct metric, it serves as a valuable tool for evaluating an algorithm’s performance. Based on the confusion matrix, various performance metrics can be derived. It provides detailed insights into the types of classification errors made by the model, which is crucial for understanding how the classifier handles different cases, such as false positives and false negatives.

### 2.3. Measurement System

After the analysis of wind turbine damage, it was decided to simulate the detection of blade damage using available components. As a prototype, small propellers and a BLDC motor were used. The vibration signal was utilized for the detection and classification of damages, which, as described in Section 1.1, are sufficient for identification of anomalous turbines operation.

**Figure 1:**
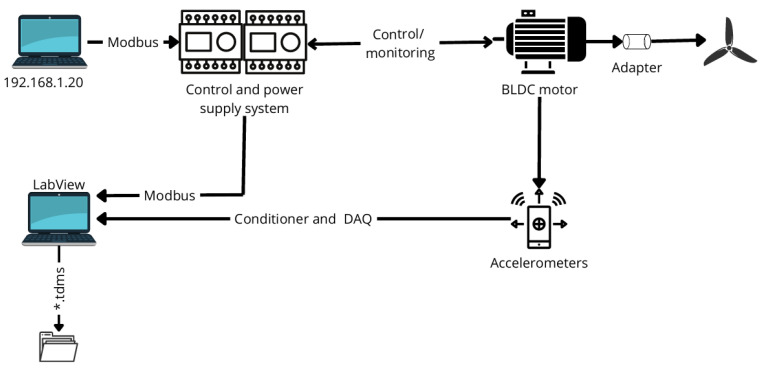
Diagram of the measurement system.

The measurements were conducted using a laboratory setup in conjunction with software implemented that enables control and data acquisition. All devices (control and power supply system, computer with a user interface enabling motor control, and computer with LabView software) are located on the same subnet (Figure 1).

An adapter to mount drone propellers with a 5 [mm] diameter hole on the BLDC motor shaft was created (Figure 1). The adapter model was designed using *Inventor 2021* software and then 3D printed. To collect vibration measurements, sensors were attached to the motor using beeswax and strong magnets. It was necessary to attach the accelerometers to be stationary to ensure that their displacement would not disrupt the study. The measurements were conducted using two Integrated Circuit Piezoelectric (ICP) accelerometers:

- single-axis (model 623C01):
  - sensitivity: (±5%) 100 mV/g (10.2 mV/(m/s^2^)),
  - frequency range: (±3dB) 48 to 900,000 cpm (0.8 to 15,000 Hz),
  - measurement range: (±50g) ±490 m/s^2^,
- three-axis (model 604B31):
  - sensitivity: (±20%) 100 mV/g (10.2 mV/(m/s^2^)),
  - frequency range: (±3dB) 30 to 300,000 cpm (0.5 to 5000 Hz),
  - measurement range: (±50g) ±490 m/s^2^.

The signal from the sensors was then passed through conditioners to the data acquisition card (DAQ) NI-9215 with the following parameters:

- number of analog input channels: 4,
- analog input voltage range: ±10 V,
- analog input resolution: 16 bits,
- maximum sample rate: 100 kS/s/ch.

The conditioner is responsible for converting the signal from the sensors into a readable signal via standard analog input devices—it converts the charge at the output of the sensors into voltage. Using the program developed in LabVIEW, data collected from the measurement card are then saved to binary files TDMS with headers.

**Figure 2:**
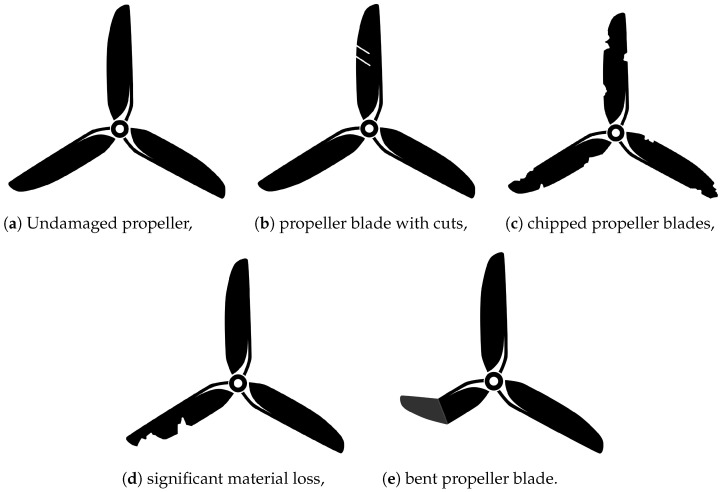
Examined propeller damage.

Five measurements were conducted separately for each degree of damage (Figure 2):

- undamaged propeller,
- propeller blade with cuts,
- chipped propeller blades,
- propeller with significant material loss,
- bent propeller blade.

This study was carried out in consecutive 15-min time intervals for speeds of 100, 200, and 300 [RPM] for approximately 5 min for each speed. The rotation was continuous in one direction. The sampling frequency for reading data from the accelerometers was 30 [kHz].

### 2.4. Data Analysis

**Figure 3:**
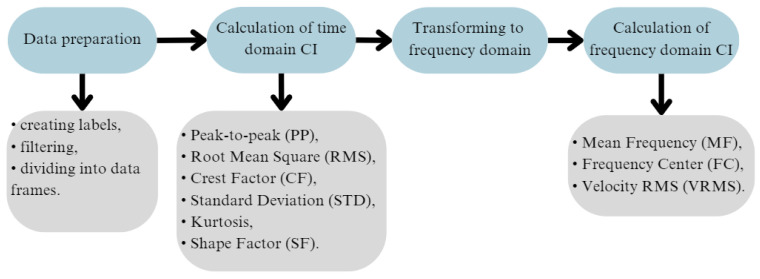
Diagram of conducted data analysis process.

The measurements ended with the acquisition of five files with the *.tdms extension. These files served as the basis for the comprehensive data analysis process, which was carried out using Python. The measurements were analyzed to gather as much valuable data as possible from the vibration signals. The complete data analysis process is presented in Figure 3.

Before calculating the diagnostic indicators, additional data processing was required. Labels corresponding to each type of damage were created and assigned to individual data points (Table 1). The signal from the three-axis sensor was filtered using a low-pass filter with a cutoff frequency $f_{c}$ = 5 [kHz] to retain only the effective measurement range for the sensor. A fifth-order Butterworth filter from the pdm_tools library was used. The next step was to divide the data into frames containing 3000 samples, corresponding to a frame duration of $t_{f}$ = 0.1 [s], with a sampling frequency $f_{s}$ = 30 [kHz]. As a result of this operation, approximately, 9000 frames were obtained for each of the five examined levels of damage.

Based on the prepared data, all selected indicators in the time domain were calculated. Next, the frames were transformed from the time domain to the frequency domain, and the frequency domain indicators were calculated (Section 2.1).

### 2.5. Machine Learning Models

Machine learning is a field of artificial intelligence consisting of a set of techniques based on statistical data analysis. It involves programming a computer in such a way that it can “learn” from data and make decisions based on provided examples. Instead of following predefined rules, machine learning algorithms analyze patterns, build models, and improve their predictions or actions with the number of training points.

This research includes the usage of three ML algorithms: random forest, support vector machine and voting classifier. All of them have been given the same task of detection and classification of propellers faults based on the scalar condition indicators. For this task, the supervised learning technique was considered—the models were trained on dataset where the expected outcomes, called labels, were known).

#### 2.5.1. Random Forest

A random forest is a collection of individual decision trees. It has all the parameters of the trees that control their size and shape. It uses the ensemble learning method to provide more stable and probable prediction. The technique involves combining multiple models to achieve better performance than a single algorithm and to minimize the risk of overfitting. Ensemble learning is particularly effective for complex problems where a single model may struggle to capture intricate relationships. It is possible to create sets from many identical or different elements [65]. Ensemble learning algorithms operate by training multiple component models on random subsets of data, then selecting the most probable prediction. The classifier can be based on majority voting (hard voting) or, if all units can compute the probability of prediction, on weighted votes based on the probability returned by each of the models (soft voting). Selection of a random subset typically occurs using the bagging method (bootstrap aggregating) with the max_samples parameter set to the size of the test set [10]. This technique involves sampling the original dataset with replacement, meaning that one instance may be selected multiple times, while another may not be selected at all.

One drawback of the random forest classifier is its high variance error (the model is excessively sensitive to small variations in the training data). A slight change in the training set can lead to the development of a completely different tree (due to the hierarchical structure of the algorithm). Mistakes in the initial nodes, close to the root of the tree, are easily propagated all the way to the leaves (nodes without any branches) [66]. Consequently, the random forest algorithm introduces additional randomness during the development of individual trees: instead of seeking globally the best value for node splitting, it searches among a random subset, what results in greater diversity among trees. To train a single decision tree, the Classification And Regression Tree (CART) algorithm is being used. It splits the training set in two subsets using a single feature k and a threshold $t_{k}$ that produces the purest subsets. The algorithm tries to minimize a CART cost function for classification given by Equation (14).

(14) $$J\left(k,t_{k}\right)=\frac{m_{left}}{m}\cdot G_{left}+\frac{m_{right}}{m}\cdot G_{right}$$

where:

$G_{left/right}$—measurement of impurity of the left/right subset,
$m_{left/right}$—the number of instances in the left/right subset [10].

Random forests, despite their simplicity, remain one of the most powerful machine learning algorithms and are widely used in many systems for both classification and regression purposes. One of their many advantages is the lack of necessity for preparing input data; they do not require scaling or centering. They cope well with a large number of dimensions and are easy to interpret. Another useful feature is the ability to measure the influence of each input parameter on the outcome.

The decision tree classifier class has several parameters that similarly restrict the shape of the tree [10]:

1. max_depth (the maximum number of nodes “levels” in the tree),
2. min_samples_split (the minimum number of samples a node must have before it can be split),
3. min_samples_leaf (the minimum number of samples a leaf node must have),
4. min_weight_fraction_leaf (same as min_samples_leaf, but expressed as a fraction of the total number of weighted instances),
5. max_leaf_nodes (maximum number of leaf nodes),
6. max_features (maximum number of features that are evaluated for splitting at each node).

**Figure 4:**
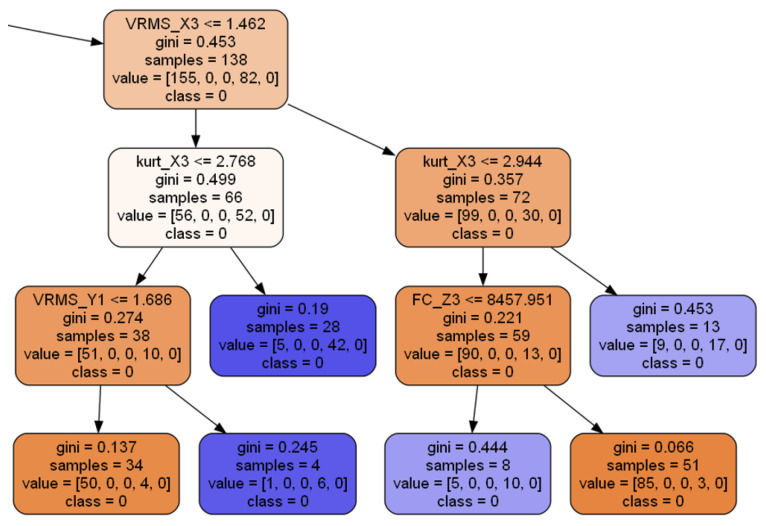
Single decision tree: closeup.

All of these parameters have a similar impact—increasing min_ hyperparameters or reducing max_ hyperparameters will regularize the model. The random forest classifier inherits every decision tree parameter but can additionally influence the number of individual trees via n_estimators. A fragment of an example decision tree is presented in Figure 4.

A node’s samples attribute counts of how many training instances it applies to; the value attribute reflects how many training instances of each class this node applies to (Figure 4). As mentioned earlier, RF can serve as a tool to measure the influence of individual input parameters on the output (by analyzing how much the nodes that are using a particular feature reduce ambiguity [67]). This impurity is represented by the gini parameter of the decision tree presented in Figure 4 and Equation (15). The node is considered “pure” if all training instances it applies to belong to the same class.

(15) $$G_{i}=1-\sum_{k=1}^{n}p_{i,k}^{2}$$

where:

$p_{i,k}$—ratio of class k instances among the training instances in the *i*th node [10].

#### 2.5.2. Support Vector Machine

Support Vector Machines (SVMs) are a powerful and versatile model that is capable of performing linear and nonlinear classification, regression, and outlier detection tasks. SVM models operate particularly well on complex but small datasets [10].

The SVM classifier can be envisioned as the widest possible path between two classes. Points located on the boundary, called support vectors, determine the path’s boundaries. This is known as *hard margin classification*, where none of the points lie beyond the boundary line. However, this method has its drawbacks: it works well only for datasets that can be linearly separated, and it is sensitive to outliers. A more flexible approach is *soft margin classification*, where some training instances may lie beyond the boundary line. The aim is to balance the maximal width of the path and minimize the number of margin violations—this trade-off is controlled by the *C* hyperparameter [68].

SVMs are powerful classification algorithms, but they have some limitations: they does not return class membership probabilities by default, and they sensitive to the scale of input values. Although linear SVM classifiers are effective and perform surprisingly well in many cases, a large portion of datasets is not linearly separable. To address nonlinear problems using a linear classifier, a technique called the “kernel trick” is employed (thoroughly explained in [10]). It involves adding more features to the data (e.g., polynomial features or similarity features) without significantly slowing down the model, as the features are not actually added. However, one disadvantage of SVMs should be mentioned: the difficulty in choosing a “good” kernel function [69]. For the solution of this particular problem, a *Radial Basis Function* (RBF) kernel was used. It uses a Gaussian function to calculate the similarity between each instance and a particular landmark, allowing for complex transformations of the data. The Gaussian RBF is given in Equation (16). It is a bell-shaped function varying from 0 (instance far away from the landmark) to 1 (at the landmark). Landmarks are selected at the location of every instance in the dataset. This creates many dimensions and thus increases the chances that the transformed training set will be linearly separable [10].

(16) $$\phi_{y}\left(x,\ell \right)={exp(-\gamma \|x-\ell \|}^{2})$$

**Figure 5:**
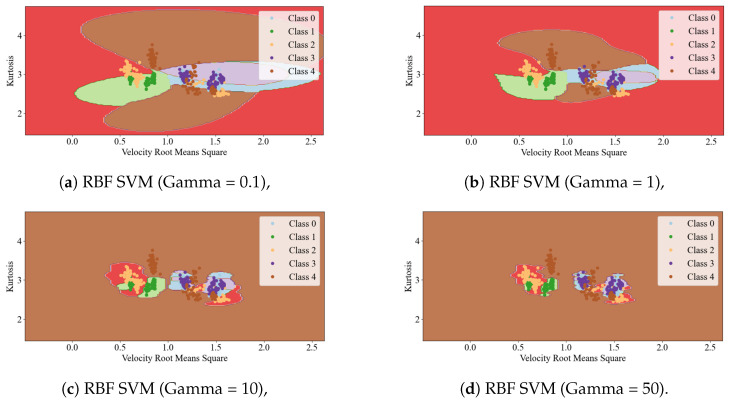
Influence of gamma parameter in SVM kernel RBF model.

The shape of the curve is controlled by the γ hyperparameter. Increasing it makes the bell-shape function narrower, and as a result, each instance’s range of influence is smaller, and the decision boundary ends up being more irregular. A small γ value makes the curve wider, instances have a larger range of influence, and the decision boundary ends up smoother. The relationship between the boundaries of the classifier and the γ value is presented in Figure 5.

In reality, the SVM classifier is a binary classifier. Therefore, to distinguish between multiple classes, the *One-vs-One* (OvO) strategy is employed. This method involves training each binary classifier to distinguish only between two classes. For K classes, it requires training and storing $\frac{K\cdot (K-1)}{2}$ different binary classifiers, which can be problematic for large values of K. Nevertheless, in the case of SVM, OvO performs significantly better than *One-vs-All* (OvA) [68] since SVMs scale poorly with the size of the training set—it is faster to train many classifiers on small training sets than training few classifiers on large training sets. Nevertheless, OvO methods are continuously being developed to improve their performance on large datasets [70].

#### 2.5.3. Voting Classifier

**Figure 6:**
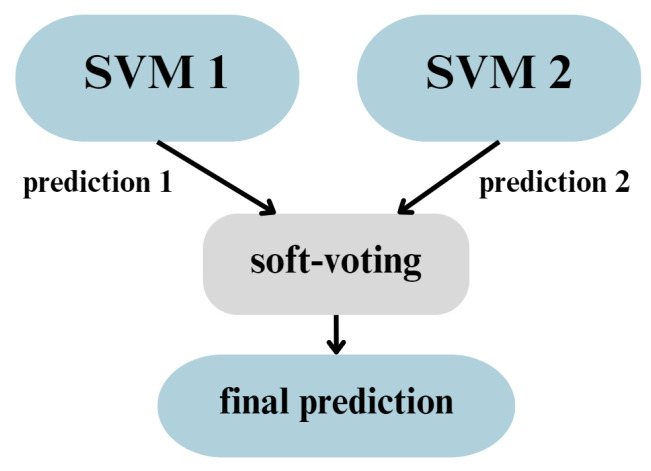
Voting classifier operation diagram.

A Voting Classifier (VC) is an ensemble learning technique used in machine learning to improve predictive performance by combining multiple models. Instead of relying on a single algorithm, the voting classifier aggregates the predictions of several different classifiers to make a final decision. This approach helps to reduce the variance and bias that individual models might introduce, leading to more accurate predictions. The EL (ensemble learning) algorithm is more precisely explained in Section 2.5.1 when describing of random forest classifier. VC can be based on majority voting (hard voting) or weighted votes based on the probabilities returned by each model (soft voting). Soft voting is considered to be more versatile; however, it is required that all componential models have the ability to return the probability of prediction. To meet the goals of this study, only machine learning algorithms were used as components, emphasizing their broad applicability. For this task, two SVM classifiers were chosen—in order to use soft voting, returning of predictions probabilities were enforced. Figure 6 presents the operating diagram of the considered VC.

### 2.6. Neural Network Classifiers

Artificial neural networks are powerful and versatile tools that are well-suited for solving large and complex problems, including both classification and regression tasks. ANNs are composed of interconnected units called neurons, each of which contains weights and biases. During training, these weights and biases are iteratively adjusted to minimize the error between predicted and actual outputs. Once trained, the neural network, with its optimized parameters, can generate accurate predictions based on input data.

In this study, two neural network models were trained to classify the state of the machine and compared with traditional machine learning solutions. The first model is a feed-forward deep neural network, while the second is a 1D convolutional neural network. Both models are known for their strong performance in classification tasks, though they differ in architecture and the way they handle input data.

#### 2.6.1. Deep Neural Network (DNN)

The architecture of the deep neural network was selected heuristically, consisting of three fully connected layers. Each layer is followed by a ReLU activation function, while a Softmax function is applied to the output layer (17). The use of ReLU introduces non-linearity, which prevents the gradient from vanishing or saturating, thereby improving the learning process. In the final layer, the softmax activation function ensures that the output values sum to 1, with each value representing the confidence level for its respective class. To control model complexity, a progressive reduction technique was used to determine the number of neurons in each layer. The first layer contains 124 neurons, the second 64, and the third 32. This gradual reduction in neurons helps to decrease the total number of parameters, which often leads to better generalization. The total number of trainable parameters in the model was equal to 14,833.

(17) $$\begin{array}{cc} h_{1} & =ReLU(W_{1}x+b_{1})\\ h_{2} & =ReLU(W_{2}h_{1}+b_{2})\\ h_{3} & =ReLU(W_{3}h_{2}+b_{3})\\ y & =Softmax(h_{3}) \end{array}$$

where:

$h_{i}$—the output of the i-th hidden layer,
*x*—the input vector,
$W_{i}$—the weight matrix for the i-th layer,
$b_{i}$—the bias vector for the i-th layer,
*ReLU*—the rectified linear unit activation function,
*softmax*—the softmax activation function,
*y*—the final output after softmax activation function.

#### 2.6.2. Convolutional Neural Network (CNN)

The second proposed solution is a 1D CNN, optimized for use with raw vibration data in the task of bearing fault identification [71,72]. The network architecture was inspired by models designed for the classification of time-series signals [73].

The optimized CNN consists of five convolutional blocks (Equation (18)), with each block containing a 1D convolutional layer using 88 filters and a kernel size of 5. Each convolutional layer is followed by batch normalization and ReLU activation. The convolutional blocks are stacked and followed by a 1D global max pooling (GMP) layer, with a Softmax layer at the output to perform the classification task (19). The pooling layer produces a feature map corresponding to each class, performs global max pooling on these maps, and passes the resulting values to the Softmax layer. As in the DNN, in the final layer, the softmax activation function ensures that the output values sum to 1, enabling each value to represent the confidence level for the respective class. The total number of trainable parameters in the model was equal to 157,085 and only 880 were non-trainable.

(18) $$\begin{array}{cc} y & =W⊗x+b\\ s & =BN(y)\\ h & =ReLU(s) \end{array}$$

where:

*W*—the weight vector,
*x*—the input,
*b*—the bias,
*s*—the normalized output using batch normalization,
*h*—the final output after ReLU activation.

(19) $$\begin{array}{cc} h_{1} & =Block_{1}\left(x\right)\\ h_{2} & =Block_{2}\left(h_{1}\right)\\ h_{3} & =Block_{3}\left(h_{2}\right)\\ h_{4} & =Block_{4}\left(h_{3}\right)\\ h_{5} & =Block_{5}\left(h_{4}\right)\\ p & =GMP_{1D}\left(h_{5}\right)\\ y & =Softmax(p) \end{array}$$

where:

*x*—the input,
$h_{1-5}$—the intermediate feature maps obtained after passing through each block in the model,
*p*—the output after global max pooling operation (GMP),
*y*—the model output obtained by applying softmax.

## 3. Condition Indicator Reduction

Feature reduction, while carrying the risk of losing certain information and potentially degrading system performance, allows us to simplify models’ architectures. In some cases, reducing the number of parameters can also filter out noise and unnecessary details, resulting in higher effectiveness. Limiting dimensions to 2 or 3 allows for clear data visualization and often leads to significant insights on the process.

Additionally, high-dimensional datasets are prone to very sparse distributions: most of the points will be far away from each other. Naturally, this also means that a new data point is likely to be similarly far from previous ones, reducing the reliability of prediction. This phenomenon is known as the curse of dimensionality, described in [10]. In summary, the more dimensions a training set has, the greater the risk of overfitting. One solution to this problem is to increase the number of data points to achieve sufficient density, but in practice, the number of points needed to achieve a given density grows exponentially with the number of dimensions [10].

**Figure 7:**
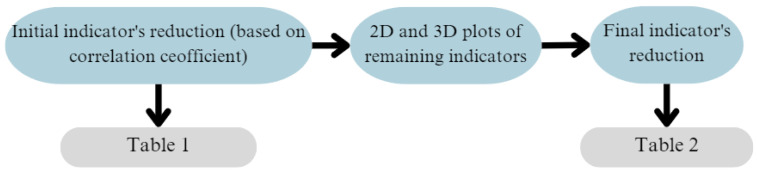
Diagram of indicator reduction process.

A diagram of the conducted condition indicator selection is presented in Figure 7.

**Figure 8:**
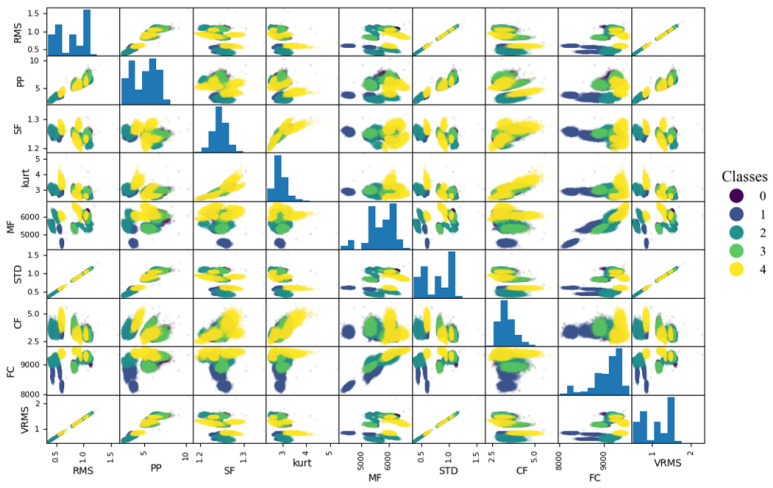
Diagram of indicator reduction process.

**Figure 9:**
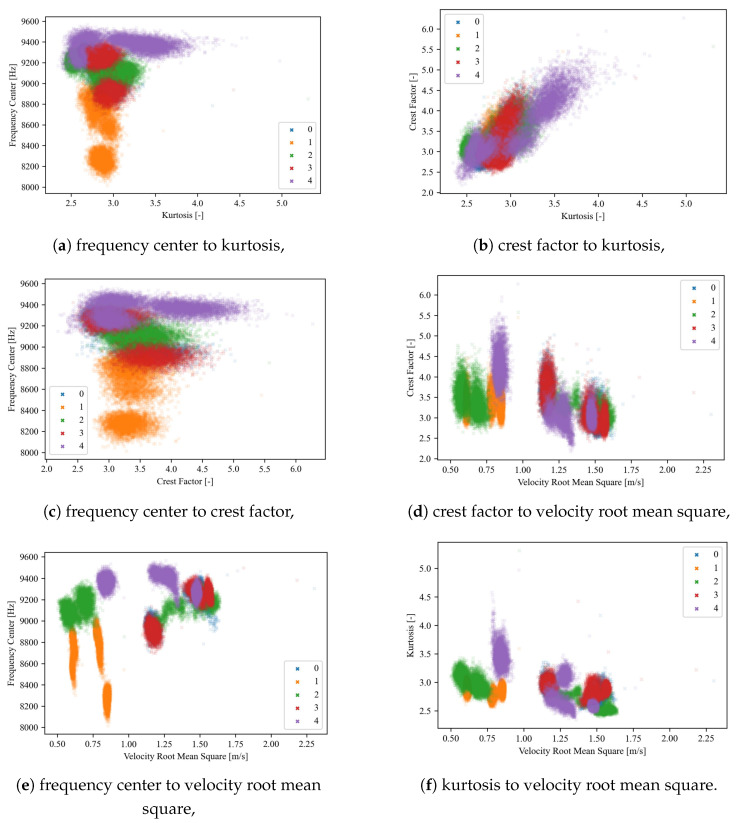
Two-dimensional plots of the relationships between indicators for the X-axis of the 3-axis accelerometer.

**Figure 10:**
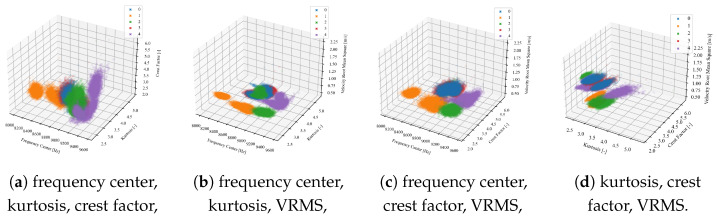
Three-dimensional plots of the relationships between indicators for the X-axis of the 3-axis accelerometer.

The proposed FS method consists of two steps (Figure 7):

- Initial selection—based on the Pearson correlation coefficient calculated between each of the preliminary indicators (Table 2). It consisted of the following substeps:
  (i) Visual analysis of relationships between indicators—before relying on Pearson’s correlation, we examined scatter plots (Figure 8) to verify that the relationships between our indicators appeared approximately linear.
  (ii) Calculation of correlation coefficients—two types of correlation coefficients were calculated, Pearson’s and Spearman’s, to additionally prove the absence of nonlinear characteristics between features (Table 3). Spearman’s rank correlation coefficient measures the strength and direction of the monotonic relationship between two ranked variables, but unlike Pearson’s correlation, which measures linear relationships, Spearman’s focuses on the ranks of the data, making it more robust to nonlinear relationships. The comparison of Table 2 and Table 3 highlights the dominance of linear and lack of purely nonlinear relationships in the data, supporting the continued use of Pearson’s correlation for initial feature selection.
  (iii) Defining the threshold value range for reduction—in [74], a correlation coefficient in the range of 0.7 to 0.89 is referred as “very strong”, and above 0.9 is “near perfect”. Based on Figure 8 and Table 2, we decided to narrow this range to exclude only very correlated features, as a further analysis will be conducted. Based on preliminary experiments examining how varying the threshold influences both the retained features and the overall models performances, we determined that the best effectiveness was achieved when the correlation threshold was set between 0.8 and 0.9. This range ensures that we only exclude highly correlated features, leaving sufficient diversity, as a further analysis will be conducted. Setting the threshold below 0.8 resulted in the removal of many features that might still be valuable to machine learning and deep learning models, which themselves can extract advanced features from the input data. Moreover, correlation-based feature reduction serves only as an initial filtering step. Setting the threshold too high would eliminate too few strongly correlated features, reducing the effectiveness of this preliminary reduction. The selection of the possible range might depend on the feature distributions and presence of noise to assure that all indicators do not have major correlations.
  (iv) Defining a specific threshold value—after continuation of preliminary experiments in predefined range, it was observed that the best threshold value of correlation was equal to 0.82 in this case. This allowed to exclude only strongly correlated features while also maintaining sufficient diversity across all four signals. While slight variations in the threshold did alter the feature set, the model’s performance remained relatively stable beyond a certain point. The selection of a specific value (0.82) within the predefined range (0.8 to 0.9) was somewhat arbitrary and is not meant as a universal guideline; rather, it should be tested on a specific dataset.
  (v) Final decision—in the case of strongly correlated indicators, one of them was eliminated. Furthermore, even though some features may have exhibited nonlinear relationships, they would not be excluded solely on this basis but rather left for further visual analysis.
  Table 4 presents the initially selected indicators for all signals: x-, y- and z-axes for the three-axis accelerometer and the signal from the one-axis sensor.
- Final reduction—relationships between the remaining indicators were plotted on 2D (Figure 9) and 3D (Figure 10) plots for visual analysis (for each one from four vibration signals). The presented plots correspond to the X-axis signal from the three-axis accelerometer. Visualization becomes essential here, allowing us to observe how features are distributed and allowing us to make informed and controlled decisions.

In this section, the whole process of FS is presented for only one signal, the X-axis of the three-axis accelerometer, but actually, it was conducted separately for each of the four measurement signals, analogically to the presented example.

Each color on the plot corresponds to a different type of the five damage levels. The formation of clusters of point clouds allows for clear differentiability of faults. Ideally, each color would form separate, tight, circular clusters, without mixing with, or even touching, other classes. On each subplot, every color forms several separate clusters, which correspond to different working conditions considered in this work. Through visual analysis, we can independently determine which relationships matter most, such as distinguishing between specific fault types (Table 1), or any other combination. For the purpose of this specific PdM solution, we wanted to assure the best disuingishability between class 0 (no fault) and 3 (significant material loss), which overlap most on the presented plots (Figure 9 and Figure 10). Such a mistake, where significant damage is incorrectly identified as no fault, presents a critical challenge in health monitoring of wind turbines. This method enables a more nuanced understanding of feature significance, leading to more effective CM.

Analyzing the 2D plots (Figure 9), a strong positive linear correlation between CF and kurtosis is visible (Figure 9b)—this means that the Pearson correlation coefficient for these two indicators is close to 1. Because of this, both indicators carry similar information for the PdM system, as they have a generally similar trend. In Figure 9b, none of the fault levels are distinguishable; they overlap and create one point cloud. Therefore, in the next step, a decision had to be made regarding which of the indicators should be eliminated. Comparing the plots of FC to kurtosis (Figure 9a) and FC to CF in Figure 9c, it is clearly visible that for kurtosis, they are more clustered, so the CF indicator was excluded. On the plot of FC to VRMS (Figure 9e), some clusters form longitudinal shapes, indicating that fault type 1 has a characteristic value for the VRMS indicator, while the FC indicator fluctuates, having a wide range of possible values. For plots where each point cloud is longitudinal (Figure 9d), we will scale indicators that have similar dispersions in each axis. On the 2D plots, it is impossible to distinguish between damage levels 3 and 0 (Figure 9, Table 1); however, plotting the relationships in 3D space improves the distinguishability (Figure 10). Still, Figure 9c,d seem rather flat in the z-axis, and the VRMS CI does not significantly improve the differentiability of fault types. Unfortunately, for dimensions greater than 3, a clear graphical visualization is not possible, but there is a presumption that individual defects will be even more separated.

The above analysis was conducted for each of the measurement signals. Table 5 presents the CI after reduction resulting from visual analysis. In Section 4.1, the correctness of the conclusions will be verified.

## 4. Results

The training of each of the models will be conducted on two datasets: a full set of CI and a set of indicators after final reduction (Table 5) without changing the parameters to verify whether any real improvement was registered.

To accurately evaluate the models, K-fold cross-validation was employed, where the training set was randomly divided into K subsets [75]. The training and validation procedure was repeated K times, each time with a different subset as the test set, while the remaining K-1 subsets formed the training set. The final result was the averaged performance of the model over all K trials. This method allows for better estimation of how well the model performs on different datasets, aiding in identifying potential overfitting or underfitting. The cross-validation algorithm also ensures that the proportion of points representing each class is maintained across all subsets. It is particularly useful when data are limited, as it eliminates the need to split the data into separate training and test sets, thus utilizing all points in the learning process. However, its applicability is not always possible, especially with complex models such as DNN, since it requires multiple training procedures.

### 4.1. Random Forest Classifier

For the problem presented in this work, it has been decided to select and adjust n_estimators and max_leaf_nodes. The parameters of the model were initially chosen heuristically, then, to select the best values, the grid search algorithm was employed. This algorithm tests all combinations of the specified parameters using the aforementioned K-fold cross-validation with 10 folds. The best combination of parameters was selected: ’n_estimators’: 850, ’max_leaf_nodes’: 90.

Once satisfactory parameter values were found, training of the actual model commenced. A fragment of the final architecture of an example decision tree within the random forest is presented in Figure 4.

Additionally, the ability of the RF to acquire information about feature relevance was used. This outcome was calculated automatically and can be accessed through the feature_importances_ variable. The results fully confirmed the conducted visual analysis. In total, 11 indicators remained, presented in Table 5.

**Figure 11:**
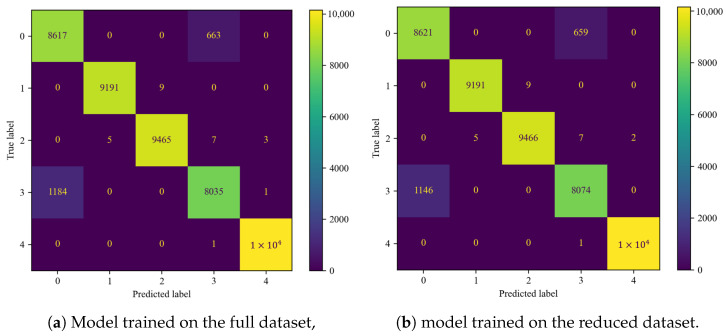
Confusion matrices for the RF classifier.

The performance evaluation was based on confusion matrices (Figure 11) and classifier metrics described in Section 2.2 (Table 6).

Comparing the confusion matrices (Figure 11a,b), a marginal increase in the distinguishability of classes 0 and 3 (Table 1) and effectiveness metrics (Table 6) is noticeable. In total, 38 fewer fault cases were misclassified as non-faulty with the reduced dataset. The rest of the matrix remained unchanged.

The metrics underwent slight but favorable changes (Table 6).

### 4.2. SVM Classifier

The same training procedure was followed for the SVM algorithm. It was also necessary to scale the input data because SVM is sensitive to differences in the scale of the input values. In the GridSearch algorithm, the values of two parameters were tested: γ and *C*. The best performance was achieved for the combination of *C* = 500 and γ = 0.1.

**Figure 12:**
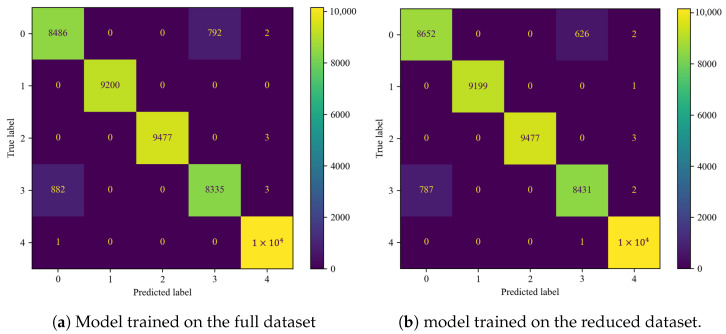
Confusion matrices for the SVM classifier.

A progression in the performance of the SVM (Figure 12a) model compared to RF (Figure 11a) is visible. Especially the misclassification of the most problematic cases (recognizing damage 3—large defect as no fault—0 (Table 1)) saw significant improvement. The remaining damages were classified with almost no errors. The overall effectiveness measures saw a slight improvement (Table 6 and Table 7).

Again, FS resulted in better distinguishability of problematic cases (Figure 12b). The numerical values of the model’s effectiveness were also improved (Table 7).

### 4.3. Voting Classifier

**Figure 13:**
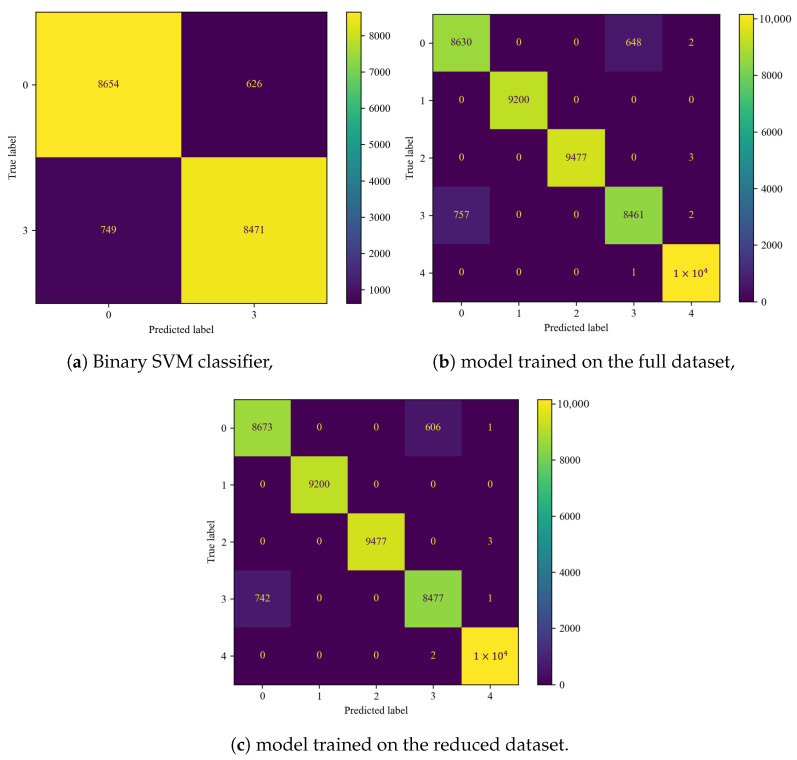
Confusion matrices for the VC classifier.

In each of the aforementioned cases, there was an issue with distinguishing between the absence of damage and a blade with a large defect. It was decided to improve the achieved results by applying ensemble learning techniques. For this task, two SVM classifiers were chosen, as this model had the best results. In the first step, the hyperparameters of the binary classifier were selected by focusing on distinguishing the two problematic categories (Figure 13a). Their values were γ = 0.03 and *C* = 10. The generated confusion matrix is presented in Figure 13a. The distinguishability of fault 3 was improved by 138 cases compared to the best of the previous models trained on the full dataset—SVM (Figure 12a). This was possible by focusing on the classification of only two cases, ignoring the other fault classes. The second chosen classifier was the SVM model that performed the best, which was used with unchanged parameters. VC combines the results of multiple algorithms to make a final decision. Finally, the obtained confusion matrix is presented in Figure 13b.

The voting classifier, even on the full dataset, performed better when distinguishing between damage 0 and 3 (Table 1) compared to the most effective model—the SVM trained on the reduced dataset (Figure 12b). The effectiveness metrics of these two algorithms are comparable (Table 7 and Table 8).

Once again, reducing the number of indicators resulted in improvements in the metrics compared to the full dataset (Table 8), as well as the distinguishability of the damages (Figure 13c). This effect can now be considered a rule for ML algorithms when operating on this specific dataset.

### 4.4. Deep Neural Network (DNN)

The DNN was trained twice to assess the impact of condition indicator reduction on performance. The dataset was split into training (80%), testing (16%), and validation (4%) sets. In both training runs, the Adam [76] optimizer was employed with an initial learning rate of 0.001, which decayed exponentially by 4% every ten epochs. Early stopping with patience of 70 epochs was used to monitor validation loss and prevent overfitting. This training setup produced satisfactory results.

**Figure 14:**
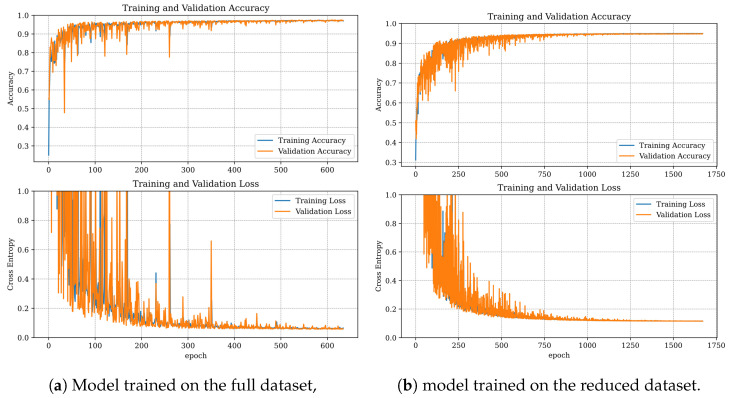
Training progress for DNN.

When trained on the reduced dataset, the network exhibited more instability and a significantly longer training duration compared to the full dataset (Figure 14). These results indicate that while the DNN learned more effectively and quickly from the full dataset, the reduced dataset still yielded good performance.

**Figure 15:**
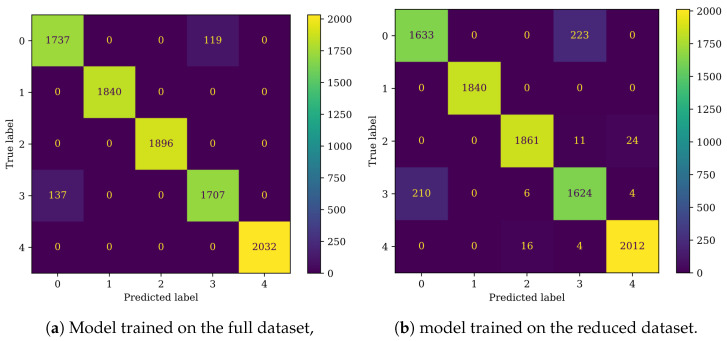
Confusion matrices for the DNN classifier.

Both DNN models performed well in the damage detection task, achieving an accuracy of 97.3% for the full dataset and 94.74% for the reduced dataset (Table 9). Other metrics, such as precision, recall, and F1 score, followed a similar distribution. Both models encountered difficulties in distinguishing between classes 0 and 3 (Figure 15, Table 1). The model trained on the reduced set of condition indicators also showed slight difficulty in classifying classes 2 and 4 (Figure 15b, Table 1), whereas the model trained on the full dataset did not exhibit these issues (Figure 15a).

### 4.5. Convolutional Neural Network (CNN)

**Figure 16:**
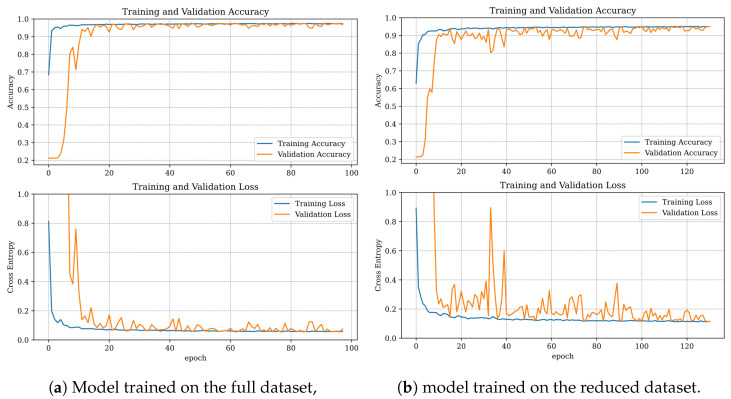
Training progress for CNN.

The training setup for the CNN was similar to that used for the DNN. The dataset was split in the same manner, and the Adam optimizer with learning rate scheduling was applied. However, there were a few key differences: the learning rate decay rate was 7%, and the patience for early stopping was set to 30 epochs. Similar observations were made during the CNN training process as with the DNN, where the model trained more stably on the full dataset (Figure 16). Unlike the DNN, the number of epochs was not significantly affected by dataset reduction.

**Figure 17:**
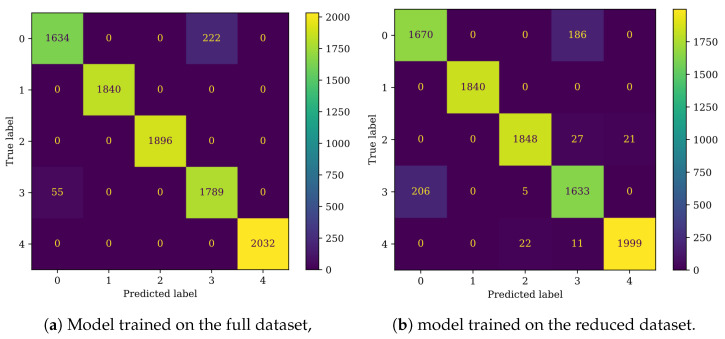
Confusion matrices for the CNN classifier.

The CNN algorithms performed very well, achieving an accuracy of 97.07% for the full dataset and 94.95% for the reduced dataset (Table 9). The performance was comparable to that of the DNN model, although the DNN slightly outperformed the CNN on the full dataset, whereas the CNN demonstrated better performance on the reduced dataset. The confusion matrices revealed that, when trained on the full dataset, the CNN model misclassified more faulty samples as healthy compared to the DNN model (Figure 17a), which was also reflected in the higher recall for the DNN. In the case of the CNN trained on the reduced dataset, the model primarily struggled with distinguishing between classes 0 and 3 (Table 1), with minor misclassifications observed in classes 2 and 4 (Figure 17b, Table 1).

### 4.6. Impact of Feature Reduction on Computational Efficiency

**Figure 18:**
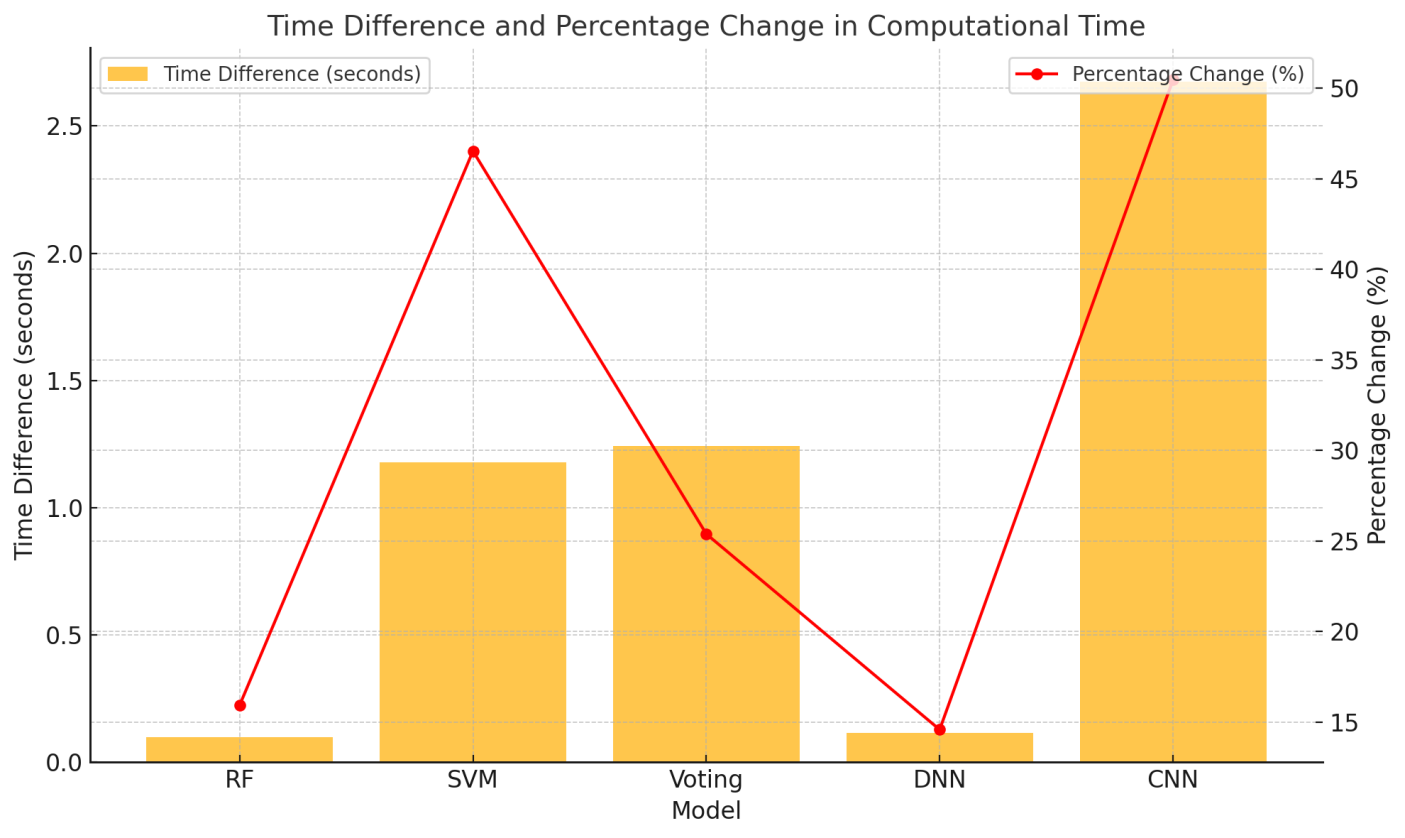
Comparison of computational time differences (in seconds) and percentage changes for various machine learning models before and after reducing the condition indicator.

To assess the impact of feature reduction on computational efficiency, we measured the average execution time for each model over 10 runs when predicting the same test batch of 9,468 samples. Reducing the condition indicator set significantly decreased the computational time required for prediction, especially in models highly sensitive to input dimensionality (Figure 18). For instance, the SVM and CNN models exhibited the most pronounced improvements, with reductions of approximately 47% and 50%, respectively, as they are particularly affected by larger datasets. Ensemble methods such as the voting classifier, along with feature-robust models like RF and DNN, also benefited from the reduced input size, showing time reductions of about 15–25%. By streamlining computations by lowering input complexity, FS not only maintains predictive accuracy but also enhances computational efficiency. This is especially critical for resource-intensive models, reinforcing the importance of careful CI selection when optimizing PdM strategies.

## 5. Discussion

In this work, a successful predictive maintenance system for the detection and classification of damages, simulating defects in wind turbine blades, has been developed. It has been demonstrated that vibration signals are a sufficient tool for accomplishing this task. Various diagnostic indicators calculated from vibrations have been presented to monitor the machine condition. In the analyzed case, a large number of these indicators are highly correlated; thus, they do not provide a significant amount of new and useful information. It was observed that ultimately, for each axis, the following diagnostic indicators were excluded:

- RMS of acceleration,
- shape factor,
- mean frequency,
- standard deviation.

Therefore, it is always worthwhile to consider feature reduction. In this study, the visual analysis perfectly aligned with the indicators’ utility calculated by the RF.

It has been proven that indicator selection plays a significant role in constructing effective PdM systems. It reduces data size, helps to avoid the problem of the curse of dimensionality, and lowers computational costs. The prediction time for every model was notably decreased, with the reduction reaching 50% for those models with the highest computational costs. Complex models can significantly benefit from the FS. Thoughtful elimination of diagnostic indicators does not negatively impact results in the case of simple ML algorithms; in fact, it improves them by eliminating unnecessary noise and details. This thesis will most often be true for measurements taken in non-laboratory conditions, where external disturbances are present. It is worth noting that for the neural networks, we can observe reversed influence of condition indicator selection. They performed better for the full set of indicators. Learning of the algorithms was also affected; on the reduced dataset, the convergence was slower and far less stable, resulting in a longer learning process. Neural network algorithms are capable of selecting the most crucial features by themselves by assigning greater weights to them compared to less-relevant CI.

Several of the most popular AI algorithms were presented, as well as interesting techniques used to improve their performance (ensemble learning, kernel trick, grid search, cross-validation). None of the algorithms could be used for Neural Networks (NNs) because training them takes significantly longer. Due to their high computational complexity and time-consuming nature, parameters were chosen in a purely heuristic manner. When using less-complicated algorithms, greater control over the results is maintained, as well as less randomness. The results of “black-box” models are more difficult to interpret and explain due to the large number of parameters.

**Figure 19:**
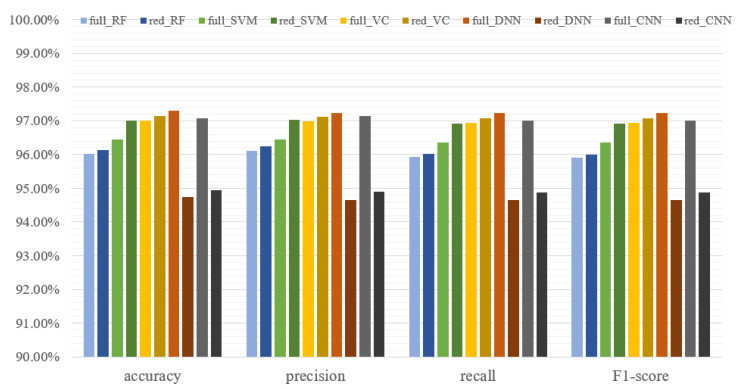
Effectiveness metrics for all models trained on reduced and full datasets.

The results of the models were presented as confusion matrices and classifiers metrics. The best results for the ML were achieved by VC (accuracy = 97.13%, precision = 97.12%, recall = 97.07%, F1 score = 97.07%). Finally, only 1.57% of all cases were falsely classified as no damage, which was prioritized in this PdM system. The best performance in the end was presented by the DNN trained on the full dataset, and the worst was for the DNN trained on the reduced dataset (Figure 19). The DNN trained on the full dataset achieved an accuracy equal to 97.3%, outperforming the VC classifier and CNN trained on the full dataset (accuracy 97.07%). However, the proposed VC solution achieved nearly the same accuracy and precision when the condition indicator selection was applied. This proves that the proposed method of selecting the condition indicators for ML algorithms is useful and can bring benefits for the designed PdM solution.

## 6. Conclusions

The proposed method to reduce condition indicators successfully reduced the number of relevant indicators for ML algorithms from 36 to 11; a reduction of approximately 3.27 times. This substantial reduction demonstrates the ability of the method to streamline data inputs without compromising diagnostic accuracy. By reducing the number of features, the proposed method allows us to reduce the complexity of the models. Furthermore, our results show that this method can significantly reduce prediction times, achieving up to a 50% decrease for more complex models. Because it is straightforward and adaptable, this method can be widely applied to various PdM tasks and is easily tailored to different diagnostic system requirements, leading to significant improvements in efficiency and performance.

Overall, for less-complex tasks, such as distinguishing among a limited number of classes, ML algorithms combined with targeted FS can achieve performance levels comparable to those of deep neural networks. Moreover, this approach offers improved interpretability by identifying the most influential indicators. The proposed method offers excellent control over the quality and quantity of selected indicators, addressing a limitation of existing solutions that often choose subsets of health indicators arbitrarily. This approach enables indicators to be tailored to specific requirements, remaining both simple and computationally efficient.

These findings indicate that carefully reducing the set of condition indicators can enhance ML algorithm performance while simultaneously lowering computational requirements and potentially decreasing sensor dependency. By laying the groundwork for a more structured approach to both indicator selection and ML model choice, this study points toward simpler solution design and even the potential for automated indicator and model selection. As a result, the development of cost-effective, efficient PdM solutions can be significantly accelerated.

## Figures and Tables

**Table 1:** Fault types and mapping classes.

	Fault Type
0	undamaged propeller
1	propeller blade with cuts
2	chipped propeller blades
3	significant material loss
4	bent propeller blade

**Table 2:** Pearson correlation coefficients between features and label for X−axis of 3−axis accelerometer (in color indicators above defined threshold).

	RMS	PP	SF	Kurt	MF	STD	CF	FC	VRMS
RMS	1.000	0.971	−0.442	−0.495	0.408	1.000	−0.481	0.459	1.000
PP	0.971	1.000	−0.340	−0.355	0.432	0.971	−0.341	0.482	0.971
SF	−0.442	−0.340	1.000	0.847	−0.122	−0.442	0.524	−0.107	−0.442
kurt	−0.495	−0.355	0.847	1.000	0.006	−0.495	0.748	0.059	−0.495
MF	0.408	0.432	−0.122	0.006	1.000	0.408	−0.179	0.926	0.408
STD	1.000	0.971	−0.442	−0.495	0.408	1.000	−0.481	0.459	1.000
CF	−0.481	−0.341	0.524	0.748	−0.179	−0.481	1.000	−0.081	−0.481
FC	0.459	0.482	−0.107	0.059	0.926	0.459	−0.081	1.000	0.459
VRMS	1.000	0.971	−0.442	−0.495	0.408	1.000	−0.481	0.459	1.000

**Table 3:** Spearman’s correlation coefficients between features and label for X-axis of 3-axis accelerometer (in color indicators above defined threshold).

	RMS	PP	SF	Kurt	MF	STD	CF	FC	VRMS
RMS	1.000	0.951	−0.443	−0.506	0.390	1.000	−0.554	0.446	1.000
PP	0.951	1.000	−0.363	−0.361	0.420	0.951	−0.402	0.470	0.951
SF	−0.443	−0.363	1.000	0.878	−0.185	−0.443	0.532	−0.089	−0.443
kurt	−0.506	−0.361	0.878	1.000	−0.119	−0.506	0.662	−0.039	−0.506
MF	0.390	0.420	−0.185	−0.119	1.000	0.390	−0.353	0.897	0.390
STD	1.000	0.951	−0.443	−0.506	0.390	1.000	−0.554	0.446	1.000
CF	−0.554	−0.402	0.532	0.662	−0.353	−0.554	1.000	−0.243	−0.554
FC	0.446	0.470	−0.089	−0.039	0.897	0.446	−0.243	1.000	0.446
VRMS	1.000	0.951	−0.443	−0.506	0.390	1.000	−0.554	0.446	1.000

**Table 4:** Remaining diagnostic indicators after initial reduction (highlighted in color) for each signal.

X3	RMS	PP	SF	kurt	MF	STD	CF	FC	VRMS
Y3	RMS	PP	SF	kurt	MF	STD	CF	FC	VRMS
Z3	RMS	PP	SF	kurt	MF	STD	CF	FC	VRMS
Y1	RMS	PP	SF	kurt	MF	STD	CF	FC	VRMS

**Table 5:** Remaining indicators after final reduction (highlighted in color) for each signal.

X3	RMS	PP	SF	kurt	MF	STD	CF	FC	VRMS
Y3	RMS	PP	SF	kurt	MF	STD	CF	FC	VRMS
Z3	RMS	PP	SF	kurt	MF	STD	CF	FC	VRMS
Y1	RMS	PP	SF	kurt	MF	STD	CF	FC	VRMS

**Table 6:** Comparison of metrics for the RF trained on the full and reduced datasets.

	Full	Reduced
Accuracy	96.02%	96.14%
Precision	96.12%	96.24%
Recall	95.94%	96.03%
F1 score	95.91%	96.00%

**Table 7:** Comparison of metrics for the SVM trained on the full and reduced datasets.

	Full	Reduced
Accuracy	96.44%	97.00%
Precision	96.44%	97.02%
Recall	96.36%	96.92%
F1 score	96.35%	96.91%

**Table 8:** Comparison of metrics for the VC trained on the full and reduced datasets.

	Full	Reduced
Accuracy	97.01%	97.13%
Precision	96.99%	97.12%
Recall	96.95%	97.07%
F1 score	96.94%	97.07%

**Table 9:** Comparison of metrics for the CNN trained on the full and reduced datasets.

	Full DNN	Reduced DNN	Full CNN	Reduced CNN
Accuracy	97.30%	94.74%	97.07%	94.95%
Precision	97.23%	94.65%	97.14%	94.89%
Recall	97.23%	94.64%	97.01%	94.87%
F1 score	97.23%	94.64%	97.00%	94.88%

## Data Availability

The data presented in this study are available upon request from the corresponding author.

## Abbreviations

The following abbreviations are used in this manuscript:

1D	One-Dimensional
2D	Two-Dimensional
AE	Autoencoder
AI	Artificial Intelligence
ARIMA	Autoregressive Integrated Moving Average
BPSO	Binary Particle Swarm Optimization
CF	Crest Factor
CI	Condition Indicators
CM	Condition Monitoring
CNN	Convolutional Neural Network
DNN	Deep Neural Network
FC	Frequency Center
FN	False Negative
FP	False Positive
FS	Feature Selection
IM	Induction Motor
KNN	K-Nearest Neighbour
MF	Mean Frequency
ML	Machine Learning
NN	Neural Network
OvA	One-vs-All
OvO	One-vs-One
PCA	Principal Component Analysis
PdM	Predictive Maintenance
PP	Peak-to-Peak
RBF	Radial Basis Function
RF	Random Forest
RMS	Root Mean Square
RNN	Recurrent Neural Network
SF	Shape Factor
STD	Standard Deviation
SVM	Support Vector Machine
TN	True Negative
TP	True Positive
VC	Voting Classifier
VRMS	Velocity Root Mean Square
